# Effects of sexual differentiation and *APOE* genotype on the development of Alzheimer-related pathology in female EFAD mice

**DOI:** 10.1186/s13293-026-00967-w

**Published:** 2026-08-12

**Authors:** Amy Christensen, Ana C. Valencia-Olvera, Helena Xeros, Elizabeth T. Nguyen, Marlene Lopez, Rojan Javaheri, Leon M. Tai, Mary Jo LaDu, Christian J. Pike

**Affiliations:** 1 Davis School of Gerontology, University of Southern California, 3715 McClintock Avenue, Los Angeles, CA 90089-0191 USA; 2 Department of Anatomy and Cell Biology, University of Illinois at Chicago, Chicago, IL 60612 USA; 3 Department of Biological Science, California State University Fullerton, Fullerton, CA 92831 USA

**Keywords:** Alzheimer’s disease, β-Amyloid, Apolipoprotein E, Organizational hormone actions, Sex differences, Sexual differentiation

## Abstract

Apolipoprotein E ε4 genotype (*APOE4*), the most significant genetic risk factor for late-onset Alzheimer’s disease (AD), is associated with sexually dimorphic effects whereby higher risks are generally observed in women. Sex differences can arise in part from developmental processes of sexual differentiation, which yield permanent sex-specific phenotypes that can impact adult trajectories and vulnerabilities to various conditions. We investigated the potential contributions of sexual differentiation to the female bias of *APOE4* in driving AD pathogenesis. Specifically, using the EFAD transgenic mouse model of AD that includes knock-in of human *APOE3* or *APOE4*, we examined the effects of experimental masculinization in perinatal female mice on adult patterns of AD-related pathology relative to control male and female mice. Both female sex and *APOE4* genotype were associated with increased levels of β-amyloid pathologies. Masculinization resulted in expected systemic and neural phenotypes in adult animals. The effects of masculinization differed depending upon both outcome measure and *APOE* genotype. Intermediate phenotypes were the most common response to masculinization, though select aspects of AD-related endpoints, including the numbers of mature amyloid plaques, were reduced by masculinization of *APOE4* female mice. These findings suggest that sexual differentiation in early life has modest effects on adult development of AD in females, a relationship that appeared stronger in the context of *APOE4*. The results complement prior work establishing roles of sex chromosomes and age-related depletion of sex hormones in the sexual dimorphisms characteristic of AD and provide new insights into the organizational actions of sex hormones and how these interact with *APOE* genotype.

**Supplementary Information:**

The online version contains supplementary material available at https://doi.org/10.1186/s13293-026-00967-w.

## Abstract (plain language summary)

Vulnerability to Alzheimer’s disease (AD) is affected by several risk factors. Women show higher prevalence and lifetime risks of AD than men. The most significant genetic risk factor for late-onset AD is *APOE4*, a normal genetic variation that is present in ~ 25% of the population. The AD-associated risk of *APOE4* is stronger in women such that that the most vulnerable population to AD is female *APOE4* carriers. It is unclear why *APOE4* affects women more strongly than men. Sex differences arise from several mechanisms, one of which is the developmental process of sexual differentiation that yields permanent sex differences in several features throughout the body. Here, we investigated the potential contributions of sexual differentiation to the female bias of *APOE4* in AD pathogenesis. Using a mouse model of AD with either human *APOE4* or the neutral risk variant *APOE3*, we examined how experimentally altering sexual differentiation in the early life of females affected their levels of AD-related pathology in adulthood. Altered female mice exhibited partially masculinized features as adults that generally were intermediate between normal males and females. Importantly, masculinized females that carried *APOE4*, but not those that carried *APOE3*, also showed partial reductions in select AD-related pathologies. These findings indicate that the developmental process of sexual differentiation is one of several factors that may contribute to the particularly high risk of AD observed in female *APOE4* carriers. These findings provide new insights into how biological sex and *APOE4* interact to drive AD risk, information that may foster development of personalized therapeutic strategies.

**Supplementary Information:**

The online version contains supplementary material available at https://doi.org/10.1186/s13293-026-00967-w.

## Highlights

- In the EFAD transgenic mouse model of Alzheimer’s disease (AD), the AD genetic risk factor APOE4 was associated with increased β-amyloid pathology that was further increased in females.
- Perinatal testosterone treatment in female EFAD mice resulted in permanently masculinized phenotypes on systemic and neural measures known to be sexually dimorphic.
- Masculinized female EFAD mice with APOE4 but not APOE3 genotype generally showed intermediate AD-related phenotypes between control male and female EFAD mice.
- These findings suggest that developmental sexual differentiation may yield a modest contribution to the female bias of APOE4 genotype in increasing AD pathogenesis.

**Supplementary Information:**

The online version contains supplementary material available at https://doi.org/10.1186/s13293-026-00967-w.

## Introduction

Alzheimer’s disease (AD) is associated with sexual dimorphisms that result in distinct differences in numerous features of the disease [1, 2]. The prevalence [3] and lifetime risk across ages [4, 5] are higher in women than men, though AD incidence is generally similar across sex [6] with a female bias apparent in old age [7]. Neuropathologically, women have increased tau accumulation [8] that appears even in prodromal stages of the disease [9, 10] as well as higher levels of neuroinflammation [11]. The relationships among indices of AD pathology and both the risks and levels of cognitive impairment are stronger in women [12, 13], suggesting women are more impacted by AD neuropathology than men. Collectively, prior findings indicate different vulnerabilities between men and women to AD risk and trajectories of progression.

One important sex difference is vulnerability to the most significant genetic risk factor for late-onset AD, the ε4 allele of apolipoprotein E (*APOE4*). In comparison to persons homozygous for *APOE3* allele, the most common genotype, *APOE4* carriers exhibit higher AD risk that increases from ~ 4-fold with one copy to > 10-fold with two copies [14]. Importantly, *APOE4*-associated AD risk interacts with sex such that women *APOE4* carriers generally show significantly higher risk than men [15, 16], though there is strong evidence this relationship exhibits age specificity [17]. Further, the combination of female sex and *APOE4* worsens numerous measures of AD progression [18–20]. The female bias in *APOE4*-related phenotypes may extend to a range of neural outcomes in non-demented healthy persons, including reductions in hippocampal volume [21], brain metabolism [22], cerebral perfusion [23], and functional connectivity [24]. Interestingly, some neural effects of *APOE4* carrier status have been reported in young adulthood [25] and even in childhood and infancy [26–28]. Potential sex differences in the effects of *APOE4* in early life have not been well studied, though there is evidence that *APOE4* is associated with lower intelligence scores in adolescent girls but not boys [29]. How and when sex differences develop in the association between *APOE4* and AD are unknown but have the promise of providing new insights into AD pathogenesis.

Here we examined whether the relationship between biological sex and *APOE* genotype on AD outcomes is impacted by early development. Specifically, we sought to determine the extent to which the established female bias of *APOE4* genotype on adult development of AD-related pathology is impacted by the spectrum of relative maleness – femaleness that is determined during critical developmental periods in early life. The brain and body largely undergo sexual differentiation into male and female phenotypes before and soon after birth. These developmental processes are mediated by organizational effects of sex steroid hormones in the perinatal period, primarily resulting from the presence of testosterone in males and estradiol sequestration in females [30]. Sexual differentiation not only drives sex-specific features, but also relative levels of masculine versus feminine phenotypes within each sex [31]. Significant contributions of developmental sexual differentiation to the heightened risk of females to AD have been reported in studies of human twins [32] and in a transgenic mouse model of AD [33]. Unknown is whether sexual differentiation contributes to the female bias in AD-associated *APOE4* effects. To investigate this possibility, we used the EFAD mouse model of AD, which contains knock-in of human *APOE* alleles in the presence of familial AD transgenes, to study the effect of experimental manipulation of sexual differentiation on AD-related neuropathology in female *APOE3* and *APOE4* mice.

## Methods

### Animals

Experimental mice were generated from a colony of EFAD mice, an AD transgenic model that is homozygous for knock-in of human *APOE3* or *APOE4* and hemizygous for the 5xFAD transgenes (*APOE*^+/+^, 5xFAD^+/−^) [34], from breeder mice provided by Dr. Mary Jo LaDu and Dr. Leon Tai. The EFAD colony is maintained by breeding of EFADs with EFAD non-carrier mice that have human *APOE* without the 5xFAD transgenes (EFAD-NC, *APOE*^+/+^, 5xFAD^−/−^). EFAD and EFAD-NC mice were housed at the University of Southern California under controlled temperature, a 12:12 light/dark schedule (lights on at 0600) with *ad libitum* access to food and water. All procedures were conducted under a protocol (#20798) approved by the USC Institution for Animal Care and Use Committee and under the supervision of USC veterinarians.

### Perinatal sexual differentiation

**Fig. 1:**
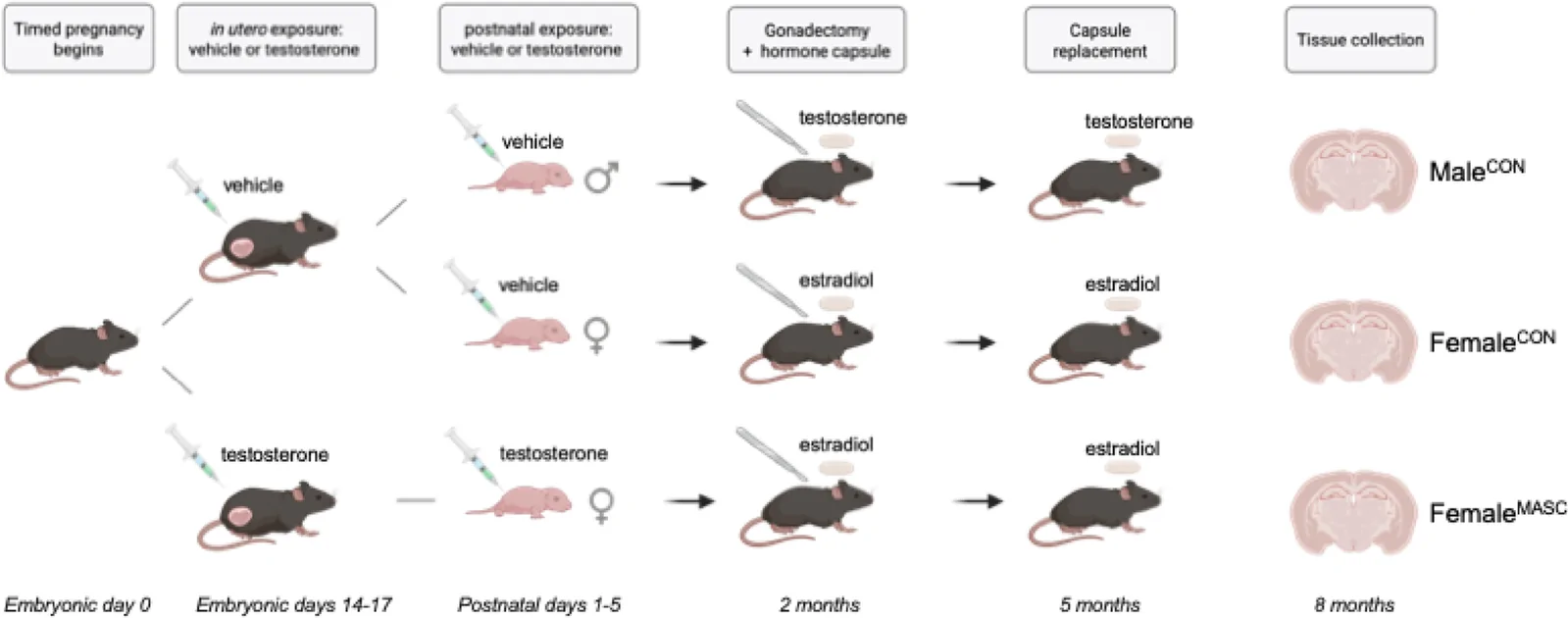
Schematic of the experimental design. Mice were time-paired and monitored for pregnancy. At embryonic days 14–17, dams were injected subcutaneously with vehicle (oil) for control male (Male^CON^) and female (Female^CON^) groups or testosterone for the masculinized female group (Female^MASC^). After pups were born, the pups were injected with the same embryonic treatment during postnatal days 1–5. At two months of age, mice were bilaterally gonadectomized and implanted with a subcutaneous hormone capsule containing testosterone (males) or estradiol (females). Three months later (age 5 months), the capsules were replaced. At 8 months of age, mice were euthanized and tissues collected for analyses

We used established methods to generate female mice with relatively masculinized adult phenotypes (Female^MASC^) that involves treatment with testosterone during critical development periods spanning *in utero* and early postnatal periods [35]. First, timed-bred pregnant dams were subcutaneously injected once daily with 0.1 mL safflower oil vehicle (for male and female controls, Male^CON^, Female^CON^) or 100 µg testosterone proprionate (TP, for Female^MASC^) on embryonic days E14-E17. All pups from a litter received a 0.025 mL subcutaneous injection of safflower oil containing 0 (Male^CON^, Female^CON^) or 100 µg TP (Female^MASC^) on postnatal days P1-P5. Thus, the Female^MASC^ group was exposed to TP both *in utero* and postnatal days P1-P5, whereas the Male^CON^ and Female^CON^ groups were only exposed to vehicle. At weaning, males and females were separated. A schematic of the treatment paradigm is provided in Fig. 1.

### Gonadectomy and capsule implantation

Female rodents that are masculinized during perinatal periods have altered levels of sex hormones in adulthood [36, 37], an outcome that can confound comparisons of adult Female^MASC^ with Female^CON^ in terms of understanding the role of early life (organizational) versus adult (activational) hormone exposures. To overcome this limitation, we equalized estradiol levels in all adult females by gonadectomy-induced depletion of endogenous ovarian hormones followed by subcutaneous implantation of a Silastic capsule containing estradiol. To control for this treatment across sex, male mice were also gonadectomized and implanted with a Silastic capsule containing testosterone. The clamping of hormone levels for all animals yields equal primary sex hormone levels in a sex-specific manner throughout late puberty and adulthood. Gonadectomies were performed at age 60 +/- 2 days using standard procedures [38, 39] that were confirmed at euthanization. All Silastic capsules (1.47 mm ID x 1.96 mm OD) were 7 mm in total length with 2 mm endcaps and 3 mm of drug delivery space. Female capsules contained 0.5% 17β-estradiol mixed in cholesterol, and male capsules had 33% testosterone in cholesterol, which were determined in house to result in uterine and seminal vesicle weights most similar to controls with limited side effects during extended treatment periods. Capsules were placed under the skin at the nape of the neck at the time of the gonadectomy surgery. All capsules were replaced a single time under isoflurane anesthesia 3 months following the initial implantation (age 5 months) (Fig. 1).

### Tissue collection

At 8 months of age, body weights were measured and mice were euthanized by carbon dioxide overdose and perfused with PBS. Brains were removed and dissected into hemispheres. One hemisphere was fixed in 4% paraformaldehyde for 72 h and transferred to PBS for subsequent use in immunohistochemistry. The other hemisphere was flash frozen on dry ice and stored at -80 °C for biochemical analyses.

### Anogenital distance

The primary measure of relative masculinization was anogenital distance [40], which was measured at the time of euthanization. The anogenital distance was defined as the length from the anus to the genitals (penis or vaginal opening), divided by the total length of the animal from the nose to base of the tail. Values were multiplied by 100 to be displayed as a percent of total body length.

### Sex behavior

Efficacy of the perinatal treatments in yielding relatively masculinized phenotypes in Female^MASC^ mice also was assessed by sex behaviors using a standard lordosis assay, which is known to be significantly reduced by perinatal masculinization [41]. Tested female EFAD-NC littermates of experimental EFAD mice received the same perinatal hormone treatments (control or testosterone) but were not gonadectomized and did not receive adult hormone capsules. At 3 months of age, females were injected with 5 µg 17β-estradiol dissolved in safflower oil 30 h prior to testing. On testing day, females were placed with a sexually experienced male and allowed to freely move within an 18 cm x 28 cm arena until they were mounted 10 times. The number of times the lordosis posture (arched back, tail moved to the side, rump uplifted) was adopted during mounting was recorded. The lordosis quotient was calculated as the percent of times lordosis was observed when the female was mounted.

### Analysis of sexually dimorphic nucleus of the medial preoptic area

Efficacy of the masculinizing perinatal treatment was assessed in a subset of animals by measuring an established brain marker of sex differences, the size of the sexually dimorphic nucleus of the medial preoptic area (SDN-MPOA) [42, 43]. EFAD-NC Male^CON^, Female^CON^ and Female^MASC^ mice at age 3 months were euthanized by carbon dioxide overdose, the brains were fixed and processed for neuronal labeling in the SDN-MPOA using immunohistochemistry for NeuN as described below. The SDN-MPOA was located using the Paxinos and Franklin mouse brain atlas (Figs. 30, 31, 32 and 33) [44] with serial coronal sections analyzed from bregma coordinates − 0.2 to 0.1 mm. Within this region, SDN-MPOA was identified as packed clusters of NeuN-labeled cells. For quantification, the cluster circumference was traced in four sections and the area determined using Image J (version 1.53) by an experienced anatomist who was blind to experimental groups. A schematic of the brain sectioning process is shown in Supplemental Fig. 1.

### Immunohistochemistry

Brains were processed for immunostaining with antibodies against NeuN, β-amyloid (Aβ), and Iba-1 using standard avidin-biotin complex methodology as previously described [45]. For Aβ and Iba-1, the fixed hemibrain from EFAD mice was sectioned exhaustively in the horizontal plane at 40 μm using a vibratome (Leica Biosystems) and sections stored singly in PBS with 0.03% sodium azide at 4 °C until immunohistochemistry was performed. Every eighth section (from a total of ~ 100 per brain) was immunostained following antigen retrieval pretreatment using 95% formic acid for 5 min for Aβ. For Iba-1, antigen retrieval was performed by boiling sections in 10 mM EDTA for 10 min. For NeuN immunolabeling, EFAD-NC brains were cryoprotected in 20% phosphate-buffered sucrose and sectioned at 20 μm on a cryostat (Leica CM1860). Four sequential sections from EFAD-NC mice containing the SDN-MPOA were processed for staining. Tissue sections were washed 3 times for 5 min in Tris-buffered saline (TBS), followed by a 10 min rinse with an endogenous peroxidase blocking solution. Next, sections were rinsed in TBS/0.1% Triton-X before being incubated for 30 min in a blocking solution consisting of TBS/2% bovine serum albumin. Sections were incubated overnight at 4 °C with a primary antibody against either β-amyloid (MOAB-2, 1:3000; Abcam, Cat #ab126649), Iba-1 (1:2,000; FUJIFILM Wako, Cat #019-19741), or NeuN (1:250; Millipore, Cat #MAB377) diluted in blocking solution. Next, sections were washed with TBS and then incubated with biotinylated anti-mouse or anti-rabbit secondary antibody (Vector Laboratories) for 1 h, followed by TBS rinses and a 1 h incubation in avidin-biotin complex (Vectastain ABC Elite kit, Vector Laboratories), and finally diaminobenzidene visualization (Vectastain DAB kit, Vector Laboratories). Stained sections were air-dried overnight, dehydrated in a series of graded alcohols, then coverslipped with Krystalon (EMD Millipore).

### Quantification of β-amyloid and Iba-1 immunoreactivities

The immunoreactive loads of Aβ and Iba-1 were quantified as previously described [45]. Briefly, the hippocampal formation was located using the Paxinos and Franklin mouse brain atlas (Figs. 141, 142, 143, 144, 145, 146, 147, 148, 149, 150 and 151) [44] with serial horizontal sections analyzed from interaural coordinates approximately 1.2 to 2.8 mm. Four sections containing the hippocampus spaced at least 320 μm apart were used for quantification. This spacing allows representation from the dorsal through the ventral regions of the hippocampus. A schematic of the brain sectioning process is shown in Supplemental Fig. 1.

For Aβ immunoreactivity, stained sections were imaged with brightfield microscopy (Olympus), imported into Image J (version 1.53) and converted to binary images. The borders of the hippocampus were manually outlined and the stained area within the hippocampal regions, subiculum or CA1, were determined for each animal using thresholding to minimize background staining. For microglial density and morphology, manual counts were performed using a 40X objective with brightfield microscopy. An 84 µm^2^ counting frame was placed over the center of the visual field. Counts of Iba-1 immunolabeled cells in the CA1 region of the hippocampus were made in two fields/section from 4 sections of each brain (8 total fields per brain). The counts were used to determine the density of microglia per mm^2^. From the same microscopic fields, Iba-1 labeled cells were characterized morphologically as previously described [46, 47]. Microglia were classified as type 1 (many thin, ramified processes), type 2 (short, thick processes and an elongated cell body), or type 3 (no or few short non-ramified processes or many filapodial processes) cells. Type 2 and 3 microglia were considered to exhibit a morphological phenotype associated with altered responsiveness.

### Thioflavin histology

To quantify levels of mature plaques, sections of hippocampus were processed with thioflavin S stain, as previously described [45]. Sections were mounted on gelatin-coated slides and allowed to air-dry overnight. The slides were rehydrated and submerged in 1% thioflavin S (Sigma) for 10 min. The stained slides were dehydrated in ethanol solutions then coverslipped with Krystalon mounting medium. The location of sections parallels the quantification of β-amyloid immunochemistry and analyses included four sections per brain that were spaced at least 320 μm apart. Images collected under epifluorescent lighting were imported into ImageJ, converted to binary images, and reversed so that plaques showed as black and the background appeared white. After thresholding to minimize background labeling, the number of plaques were measured in the subiculum and CA1 areas of the hippocampus.

### Protein extractions for biochemical analyses

Three-step-sequential protein fractionation was performed as previously described [48, 49] to yield soluble (Tris-buffered saline: TBS), non-ionic detergent soluble (TBS + 1%Triton X-100: detergent) and insoluble (neutralized formic acid: FA) fractions. Within the apoE extraction profile, apoE-lipoproteins are detected in the detergent fraction, which includes a non-ionic detergent that releases apoE from the lipoproteins without formation of new micelles as would occur within harsher, Triton-X ionic detergents. The level of apoE within the TBS-X fraction serves as a measurement of apoE lipidation. The insoluble FA fraction represents apoE-associated with Aβ plaques that require harsher solvent for extraction. Within the Aβ extraction profile, soluble oligomeric Aβ (oAβ) is largely found within the TBS fraction. The FA extraction contains insoluble Aβ released from plaques. Total protein in the TBS and FA fractions was quantified with the Ready to Use Bradford Reagent (Bio-Rad) and total protein in the TBS-X fractions with the BCA Protein Assay Kit (Pierce).

### ELISAs

Soluble oAβ levels were measured in the soluble fraction of brain homogenates (biosensis) and insoluble Aβ42 (Life Technologies) in the FA fraction. ApoE levels were measured in TBS, detergent, and FA cortex homogenates by ELISA as previously described. All samples were diluted to fit within the sensitivity of each ELISA.

*Statistics.* Two-way ANOVAs were used to determine the effects of *APOE* and perinatal treatment. Between group comparisons were determined when indicated by a significant (*p* < 0.05) main effects or interactive effects. One-way ANOVAs or *t*-tests were used when data were collapsed across *APOE* genotype (Supplemental Fig. 2). All statistical results are listed in Table 1 and Supplemental Table 1. All significant effects are listed in the tables and noted on the graphs. In addition, comparisons with nonsignificant trends with *p* < 0.10 are included in the tables.

## Results

### Efficacy of masculinization treatments

To confirm that the perinatal treatments successfully masculinized females, peripheral and neural measures of sexual dimorphism were measured. First, we measured the area of the sexually dimorphic nucleus of the medial preoptic area (SDN-MPOA), a brain region that develops larger in males than females as a consequence of sexual differentiation [50]. As expected, the SDN-MPOA area in *APOE3* and *APOE4* littermates of the experimental EFAD mice showed the expected outcome of Male^CON^ mice have significantly larger areas than Female^CON^ mice (Supplemental Fig. 2A, Supplemental Table 1). Female^MASC^ mice exhibited intermediate SDN-MPOA areas that were significantly larger than Female^CON^ mice but not significantly less than Male^CON^ mice (Supplemental Fig. 2A), indicating masculinized SDN-MPOA in the Female^MASC^ mice. The SDN-MPOA contributes to sexual behavior in rodents [41]. To confirm functional effects of sexual differentiation, female EFAD littermates were tested for sexual behavior competency. Female^CON^ mice displayed normal sexual responsiveness with an average lordosis quotient of 85, whereas Female^MASC^ displayed zero lordosis behavior when paired with a stud male despite being primed with estradiol (Supplemental Fig. 2B, Supplemental Table 1). Both measures confirm expected sex differences between Male^CON^ and Female^CON^ mice as well as efficacy of testosterone treatment in yielding masculinized phenotypes in young adult Female^MASC^ mice.

**Fig. 2:**
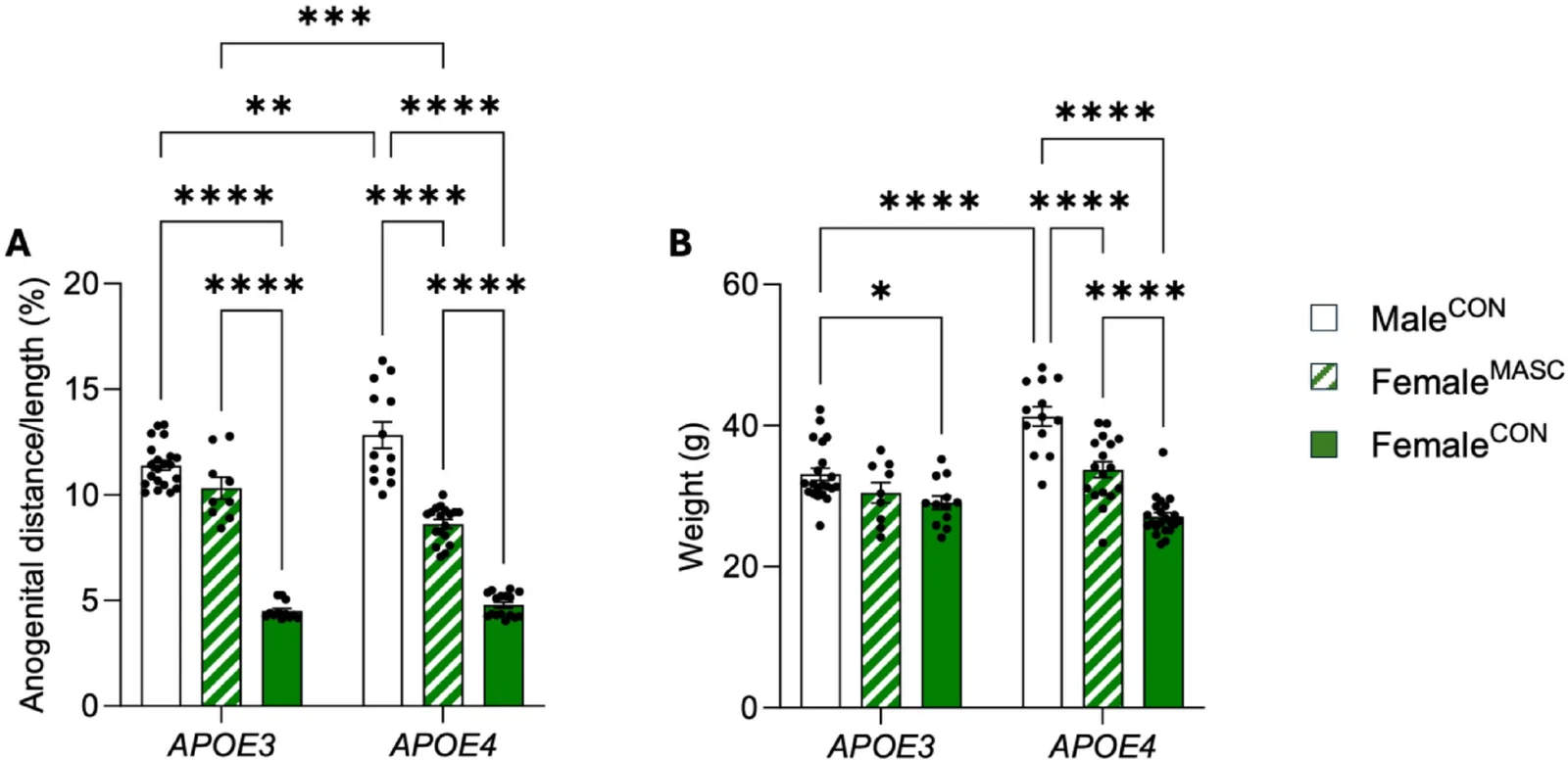
Effects of experimental treatments on adult sexual dimorphisms. Two sexually dimorphic phenotypes, (**A**) anogenital distance and (**B**) body weight, were measured at the time of euthanasia in *APOE3* and *APOE4* EFAD mice in the following treatment groups: control males (Male^CON^, open bars), masculinized females (Female^MASC^, hatched bars), control females (Female^CON^, solid green bars). Data show mean ± SEM; data points from individual mice are indicated by solid circles. Details of statistical analyses are shown in Table 1. Brackets denote significant between group differences: * *p* < 0.05; ** *p* < 0.01; *** *p* < 0.001; **** *p* < 0.0001

Measures of sexual differentiation were assessed in experimental EFAD mice at the end of the study using two additional sexually dimorphic outcomes: anogenital distance and body weight. As expected, in both *APOE* genotypes, Male^CON^ mice had a significantly greater anogenital distance than Female^CON^ mice (Fig. 2A; Table 1). Interestingly, the magnitude of the sex difference showed a statistically nonsignificant trend of being greater in *APOE4* (Male^CON^ = 267% of Female^CON^) than *APOE3* (Male^CON^ = 253% of Female^CON^) mice. Female^MASC^ mice had a significantly longer anogenital distance than Female^CON^ mice in both *APOE* genotypes (Fig. 2A), confirming a systemic masculinizing effect of perinatal treatments. However, there was a significant Treatment X Genotype interaction (Table 1) such that the masculinizing effect of testosterone treatment yielded a relatively stronger effect in *APOE3* mice. Indeed, Female^MASC^ mice significantly differed from Male^CON^ mice in *APOE4* but not *APOE3* mice (Fig. 2A).

We also assessed sexual differentiation by body weight. In both *APOE* genotypes, Male^CON^ mice exhibited significantly higher body weights than Female^CON^ mice, though the sex difference was more robust in *APOE4* (males 152% heavier) than *APOE3* mice (males 114% heavier) (Fig. 2B). Female^MASC^ mice showed intermediate body weight between Male^CON^ mice and Female^CON^ mice, however there was a significant Perinatal treatment X Genotype interaction such that the increase in Female^MASC^ mice relative to Female^CON^ mice reached statistical significance only in *APOE4* mice (Fig. 2B; Table 1). Collectively, these data confirm both the established sexual dimorphisms and efficacy of the masculinizing testosterone treatment, while also indicating *APOE* genotype-associated differences.

### β-Amyloid deposition

**Fig. 3:**
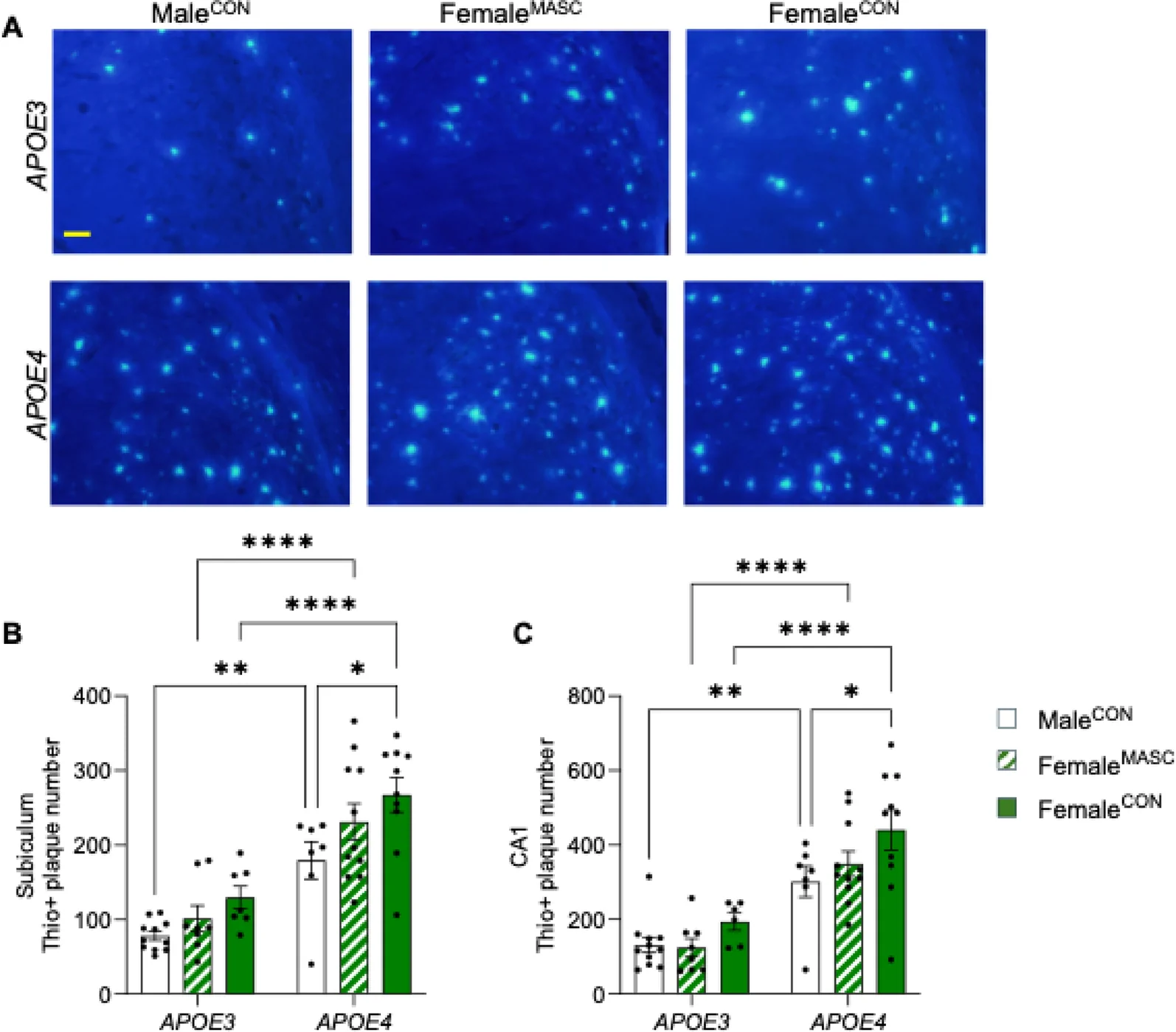
Effects of experimental treatments on amyloid plaque number. Mature amyloid plaques were assessed by quantification of thioflavin-S staining. (**A**) Representative **i**mages show brightly labeled amyloid plaques in the hippocampal formation in Male^CON^, Female^MASC^, and Female^CON^ groups from *APOE3* and *APOE4* EFAD mice. Scale bar = 50 μm. Numbers of thioflavin-labeled deposits were counted in the (**B**) subiculum and (**C**) CA1 regions of the hippocampus in control males (Male^CON^, open bars), masculinized females (Female^MASC^, hatched bars), control females (Female^CON^, solid green bars). Data show mean ± SEM; data points from individual mice are indicated by solid circles. Details of statistical analyses are shown in Table 1. Brackets denote significant between group differences: * *p* < 0.05; ** *p* < 0.01; *** *p* < 0.001; **** *p* < 0.0001

 The effects of perinatal masculinization of females on sexually dimorphic patterns of AD-related pathology across *APOE3* and *APOE4* genotypes were investigated in the hippocampal formation, a brain region that exhibits both sexual dimorphisms [51] and is highly vulnerable to AD pathology in humans [52] and the EFAD mouse model [53]. Pathological deposition of the AD-related protein Aβ was determined by two approaches. First, the number of mature amyloid plaques was determined in regions of the hippocampal formation using thioflavin-S staining. Consistent with prior findings [54, 55], Aβ plaque levels in the subiculum and CA1 regions of the hippocampus were higher in *APOE4* mice than *APOE3* mice, and this effect was stronger in females such that the highest plaques levels were observed in *APOE4* Female^CON^ mice (Fig. 3). The female bias in plaque number was statistically significant only in *APOE4* mice, with *APOE3* mice showing nonsignificant trends favoring Female^CON^ mice. If masculinization reduces Aβ accumulation, then Female^MASC^ mice would be predicted to show fewer plaques than Female^CON^ mice. Across subiculum and hippocampus CA1 in both *APOE3* and *APOE4* mice, the Female^MASC^ group showed intermediate plaque numbers that did not significantly differ from the Male^CON^ and Female^CON^ groups (Fig. 3).

**Fig. 4:**
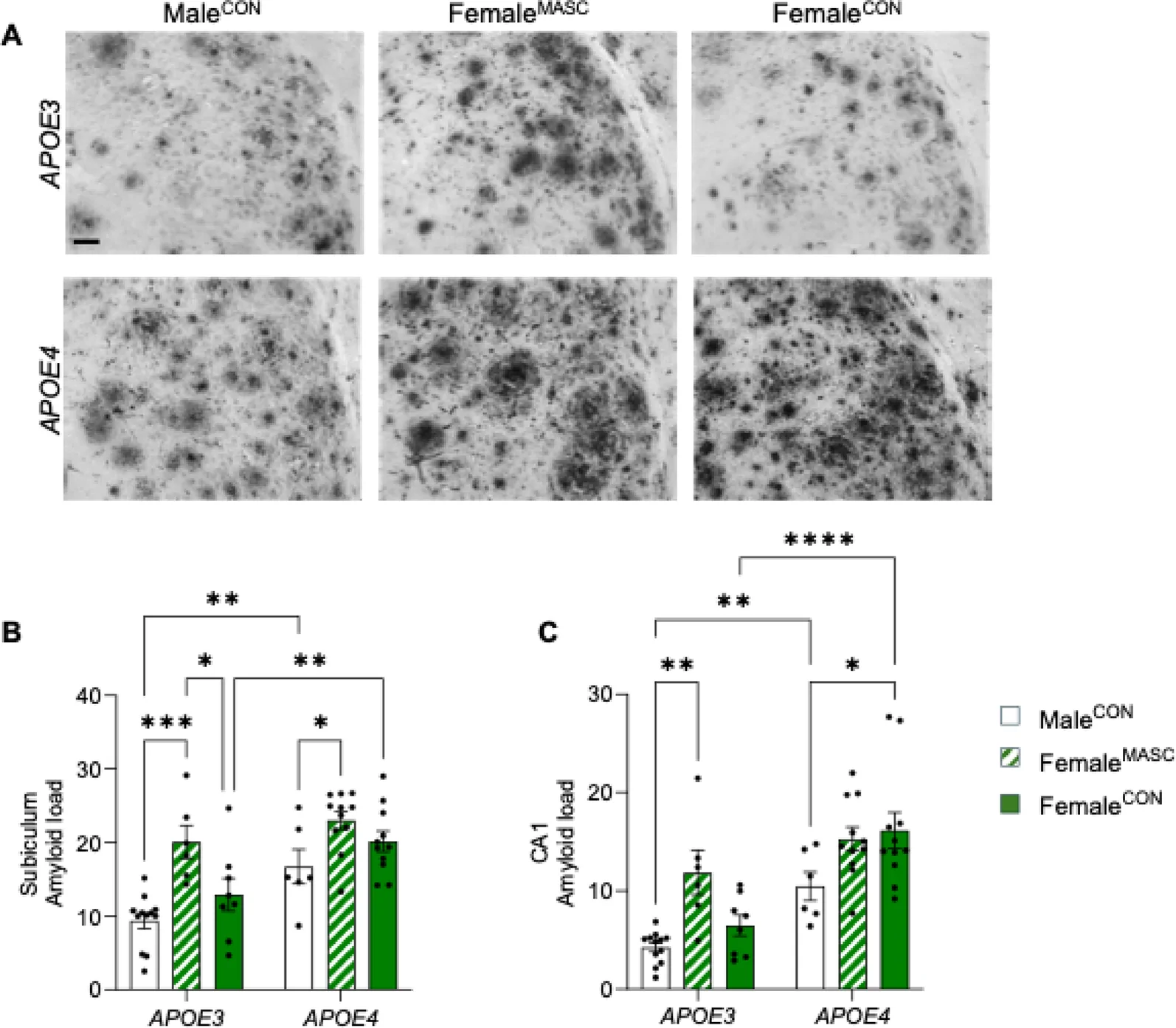
Effects of experimental treatments on β-amyloid burden. Accumulations of β-amyloid protein were assessed by quantification of β-amyloid immunohistochemical load. (**A**) Representative **i**mages show β-amyloid immunoreactivity in the hippocampal formation in Male^CON^, Female^MASC^, and Female^CON^ groups from *APOE3* and *APOE4* EFAD mice. Scale bar = 50 μm. Immunoreactive loads of β-amyloid were measured in the (**B**) subiculum and (**C**) CA1 regions of the hippocampus in control males (Male^CON^, open bars), masculinized females (Female^MASC^, hatched bars), control females (Female^CON^, solid green bars). Data show mean ± SEM; data points from individual mice are indicated by solid circles. Details of statistical analyses are shown in Table 1. Brackets denote significant between group differences: * *p* < 0.05; ** *p* < 0.01; *** *p* < 0.001; **** *p* < 0.0001

Second, we assessed total levels of Aβ deposition by quantifying Aβ immunoreactive load. As with thioflavin-positive plaque number, we observed that Aβ immunoreactive load was relatively higher in the contexts of both female sex (Female^CON^ > Male^CON^) and *APOE4* genotype (Fig. 4). However, in contrast to the plaque data, the effects of masculinization on Aβ load were not straightforward. In *APOE3* mice, Female^MASC^ mice showed the highest Aβ loads in both the subiculum and CA1 regions with a significant increase relative to Female^CON^ mice in the subiculum (Fig. 4). In *APOE4* mice, a similar relationship of higher Aβ load in the Female^MASC^ group was observed in subiculum. In CA1 hippocampus, Aβ loads were significantly higher in Female^CON^ than Male^CON^ *APOE4* mice, and Female^MASC^ mice displayed an intermediate phenotype that was not different from either sex (Fig. 4C; Table 1).

### β-Amyloid levels in brain extracts

**Fig. 5:**
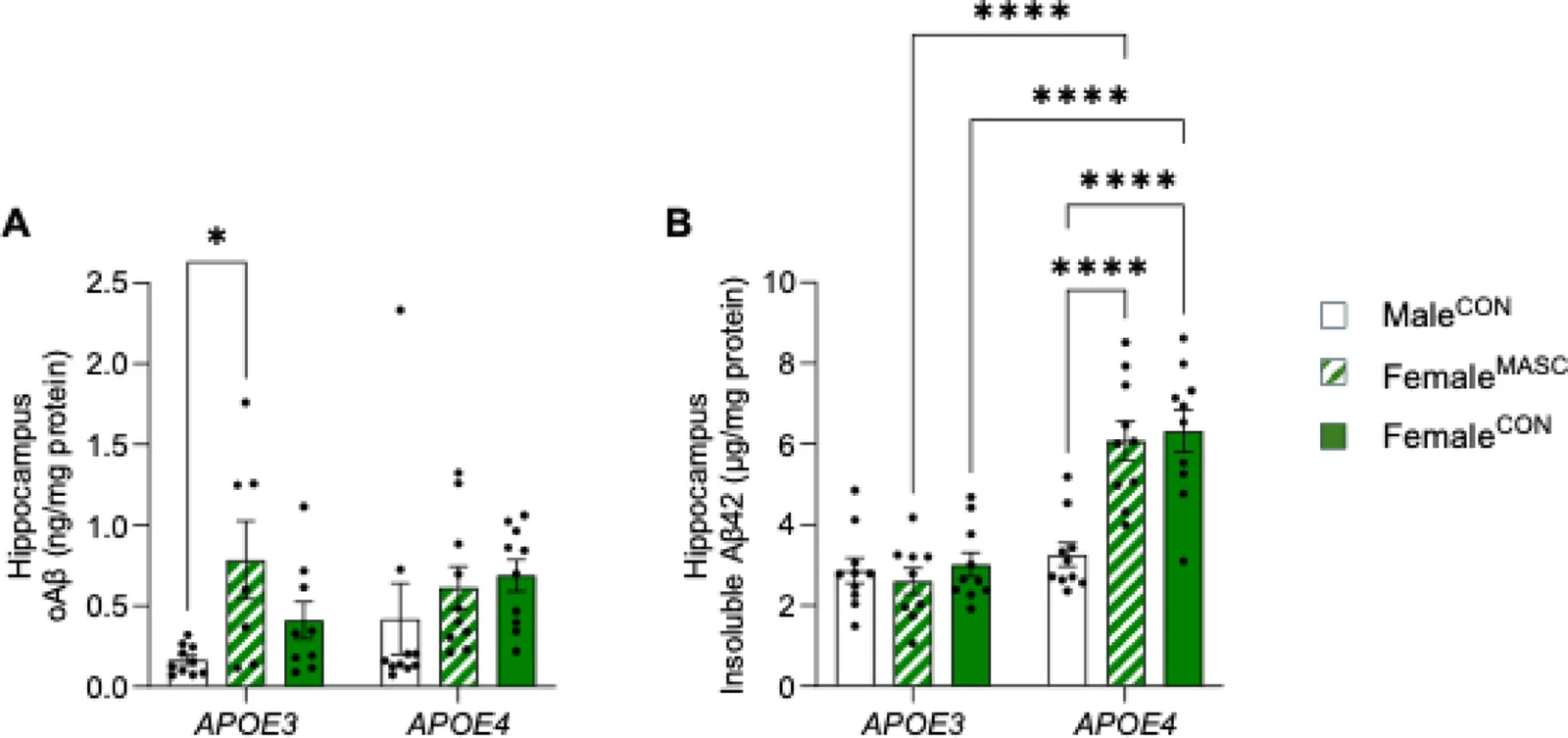
Effects of experimental treatments on levels of oligomeric and insoluble β-amyloid. Levels of the highly amyloidogenic Aβ42 species of β-amyloid protein were measured by ELISA in hippocampal homogenates. (**A**) Oligomeric Aβ42 (oAβ) was measured in the soluble fraction, and (**B**) relatively insoluble Aβ42 was measured in the formic acid soluble fraction from control males (Male^CON^, open bars), masculinized females (Female^MASC^, hatched bars), and control females (Female^CON^, solid green bars). Data show mean ± SEM; data points from individual mice are indicated by solid circles. Details of statistical analyses are shown in Table 1. Brackets denote significant between group differences: * *p* < 0.05; **** *p* < 0.0001

To gain further insight into the effects of developmental masculinization on adult Aβ accumulation, we homogenized hippocampus and extracted these into three fractions representing decreasing relative solubility: TBS extraction, detergent extraction, and formic acid (FA) extraction. First, we measured levels of oligomeric Aβ42 (oAβ) by ELISA, which is found largely within the TBS-soluble fraction. In parallel to Aβ deposition findings, levels of oAβ were generally higher with female sex and *APOE4* genotype, although these did not achieve statistical significance. There was a significant main effect of perinatal treatment group that was driven by the Female^MASC^ group specifically in *APOE3* mice, which showed the highest oAβ levels (Fig. 5A; Table 1). Next we measured Aβ42 levels in the relatively insoluble FA fraction, which strongly overlaps with the Aβ pool associated with mature plaques. There were significant main effects of both treatment group and *APOE* genotype that were driven by *APOE4* as there were no significant differences in levels of FA-soluble Aβ42 across Male^CON^, Female^MASC^, and Female^CON^ groups from *APOE3* mice (Fig. 5B; Table 1). In *APOE4* mice, Female^CON^ mice had significantly higher FA-soluble Aβ42 than Male^CON^ mice. The Female^MASC^ mice had FA-soluble Aβ42 levels nearly as high as the Female^CON^ group and significantly higher than the Male^CON^ mice (Fig. 5B). Parallel analyses of oAβ and FA-soluble Aβ42 performed in fractions of cerebral cortex yielded qualitatively similar results in which Female^MASC^ group was associated with (i) highest Aβ42 levels in *APOE3* mice, and (ii) intermediate Aβ42 levels between Female^CON^ and Male^CON^ groups in *APOE4* mice (Supplemental Fig. 3, Supplemental Table 2).

**Fig. 6:**
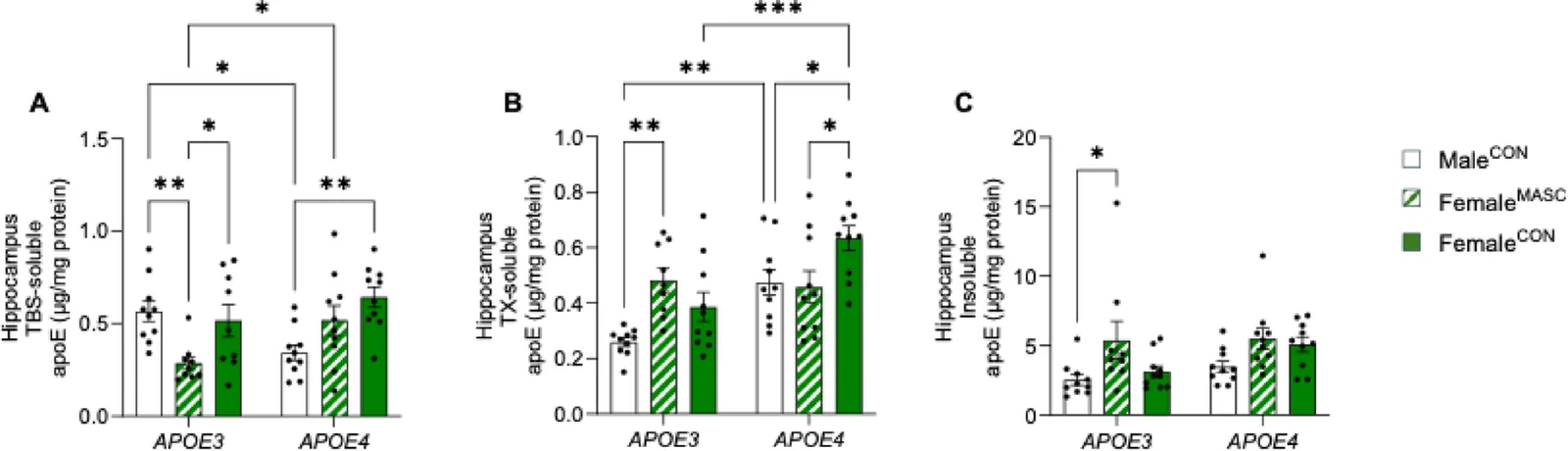
Effects of experimental treatments on levels of apoE protein. Levels of apoE were measured by ELISA in hippocampal homogenates extracted into (**A**) TBS-soluble fractions, (**B**) detergent (Triton-X100)-soluble fractions, and (**C**) relatively insoluble formic acid-soluble fractions from control males (Male^CON^, open bars), masculinized females (Female^MASC^, hatched bars), and control females (Female^CON^, solid green bars). Data show mean ± SEM; data points from individual mice are indicated by solid circles. Details of statistical analyses are shown in Table 1. Brackets denote significant between group differences: * *p* < 0.05; ** *p* < 0.01; *** *p* < 0.001

*ApoE levels in brain extracts.* Prior work has demonstrated that apoE levels in EFAD mice can vary by sex and or *APOE* genotype [53] and that they are associated with Aβ levels [48, 53, 56]. Using our three-step brain fractionation protocol [57], we compared hippocampal levels of relatively soluble apoE (TBS fraction), lipidated apoE (detergent fraction), and plaque-associated apoE (FA fraction). There was a significant main effect of treatment group on apoE levels in all three fractions (Fig. 6; Table 1). In *APOE3* mice, across all fractions, there were no statistically significant differences between Female^CON^ and Male^CON^ groups, but the Female^MASC^ group showed significant differences with Female^CON^ and or Male^CON^ groups. These data suggest an absence of strong *APOE3* sex differences though a significant impact of masculinization treatment. In *APOE4* mice, there were significant differences between Female^CON^ and Male^CON^ groups in both the TBS and detergent fractions (Fig. 6A, B; Table 1) and a nonsignificant trend in the FA fraction (Fig. 6C), indicating *APOE4*-dependent sex differences in apoE levels. The Female^MASC^ group showed intermediate apoE levels between Female^CON^ and Male^CON^ groups in the TBS fraction, and apoE levels that aligned with Male^CON^ and significantly differed from the Female^CON^ group in the detergent fraction (Fig. 6; Table 1). No group differences were observed in the FA fraction of *APOE4* mice, suggesting sex and perinatal masculinization affect apoE levels in relatively soluble but not insoluble pools. We similarly measured apoE levels in fractionated cerebral cortex and observed qualitatively similar findings with the primary difference being significant sex and treatment effects were also observed in the FA fraction of *APOE4* mice (Supplemental Fig. 4, Supplemental Table 2).

### Assessment of microglia

**Fig. 7:**
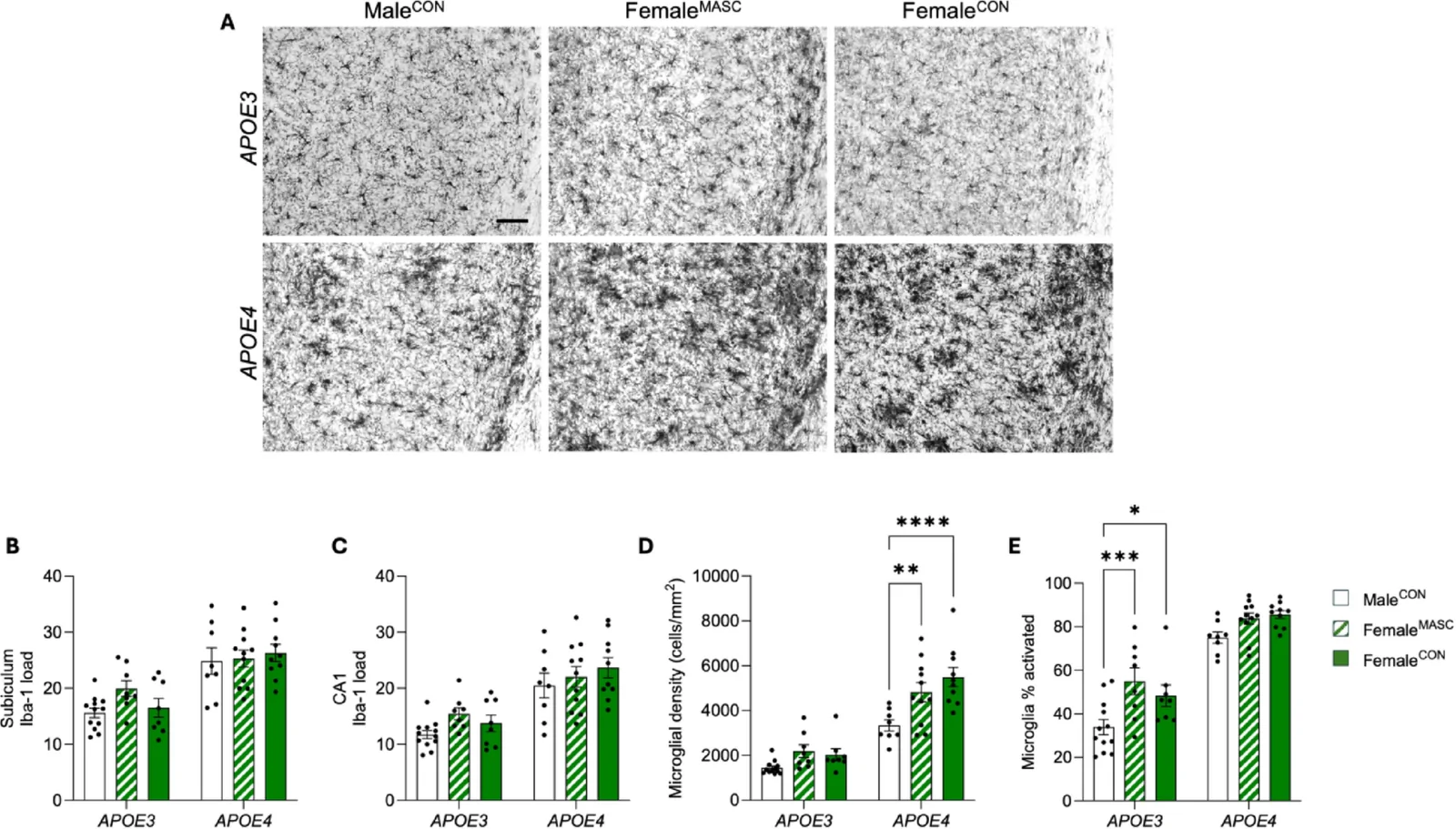
Effects of experimental treatments on microglia. Microglia were examined by Iba-1 immunohistochemistry. (**A**) Representative images of Iba-1 immunolabeling in hippocampus CA1 from control male (Male^CON^), masculinized female (Female^MASC^), and control female (Female^CON^) EFAD mice with *APOE3* (top row) and *APOE4* (bottom row) genotypes. Scale bar = 50 μm. Levels of Iba-1 immunohistochemical burden were measured in subiculum (**B**) and hippocampus CA1 (**C**). Iba-1 immunolabeled cells in hippocampus CA1 were analyzed for (**D**) relative abundance as determined by cell density, and (**E**) morphology associated with microglial responsiveness. Data show mean ± SEM from control males (Male^CON^, open bars), masculinized females (Female^MASC^, hatched bars), and control females (Female^CON^, solid green bars). Data points from individual mice are indicated by solid circles. Details of statistical analyses are shown in Table 1. Brackets denote significant between group differences: * *p* < 0.05; ** *p* < 0.01; *** *p* < 0.001; **** *p* < 0.0001

Finally, we examined abundance and morphological evidence of responsive state of microglia, a cell type implicated as a modulator of AD pathogenesis [58] that is affected by both sex and *APOE* genotype [59, 60]. Immunohistochemistry with the microglial marker Iba-1 shows readily apparent differences in the overall abundance of microglia in *APOE4* versus *APOE3* EFAD mice across sexes (Fig. 7A). Quantitative analysis of Iba-1 immunohistochemical burden confirms this effect, demonstrating significantly higher Iba-1 load in both subiculum (Fig. 7A; Table 1) and hippocampus CA1 (Fig. 7C; Table 1) in *APOE4* EFAD mice in the absence of significant sex differences. The density of Iba-1 immunolabeled cells was higher in *APOE4* EFAD mice, and significantly increased levels were observed in Female^CON^ and Female^MASC^ groups relative to the Male^CON^ group only within *APOE4* mice (Fig. 7D; Table 1). The proportion of Iba-1 immunolabeled cells that exhibit morphology associated with a responsive phenotype was approximately two-fold higher in *APOE4* versus *APOE3* EFAD mice (Fig. 7E). Further there are sex differences in which higher levels of the responsive phenotype were observed.

## Discussion

Women have increased prevalence and risk of AD as well as heightened vulnerability to outcomes associated with *APOE4*, the most significant genetic risk factor for late-onset AD. We investigated whether the female biases in AD and *APOE4* risks are mediated in part by the developmental process of sexual differentiation. Specifically, we assessed how experimentally altering female sexual differentiation towards a relatively masculine phenotype affected the adult pattern of AD-related neuropathology. We used the EFAD mouse model of AD that includes targeted replacement of mouse *APOE* with either human *APOE3* or *APOE4* to allow comparison of results by *APOE* genotype. Our data demonstrate the expected finding of higher Aβ-related pathologies with both *APOE4* genotype and female sex in which the female bias was most apparent in the context of *APOE4*. Importantly, our results also indicate a subtle moderating effect of sexual differentiation on select aspects of this relationship. Specifically, relative to Male^CON^ *APOE4* mice, significantly higher numbers of mature plaques are observed in Female^CON^ mice but a reduced, statistically nonsignificant elevation is seen in phenotypically masculinized female (Female^MASC^) *APOE4* mice. These new findings suggest a modest but significant role of sexual differentiation underlying the female bias of *APOE4* on the development of AD-related pathology.

The masculinization protocol was effective in both *APOE* genotypes as evidenced by peripheral changes that were intermediate to the Male^CON^ and Female^CON^ groups within each genotype (Fig. 2). Across the AD-related measures, however, *APOE3* Male^CON^ and Female^CON^ mice generally showed statistically nonsignificant differences. In contrast, *APOE4* mice often exhibited different trends in response to masculinization, though statistical significance was primarily observed by *post hoc* analyses of main effects rather than more robust interactions of main effects. More specifically, *post hoc* analyses demonstrated numerous significant differences between Male^CON^ and Female^CON^ *APOE4* mice on AD-related outcomes with Female^MASC^ often resulting in intermediate effects. The observed *APOE* genotype differences in sexual dimorphic AD-related measures impacts the interpretation of the results. Because significant sex differences were relatively limited in *APOE3* mice, detection of potential moderating effects of masculinization on AD endpoints was challenging in *APOE3* mice. Indeed, masculinization appeared to result in an alternative pathway that yielded phenotypes in Female^MASC^ *APOE3* mice different from both Male^CON^ and Female^CON^ groups. This unexpected finding requires further investigation.

As the protective effect of masculinization was limited to specific pathologies and largely to *APOE4* genotype, sexual differentiation likely contributes in a limited manner and or to select aspects of the relationships among female sex, *APOE4* and AD. Specifically, the significantly higher level of mature, thioflavin-positive amyloid plaques observed in Female^CON^ versus Male^CON^ *APOE4* mice was no longer present in Female^MASC^ *APOE4* mice, which significantly differed from neither the Female^CON^ nor Male^CON^ groups. A similar but statistically nonsignificant trend was observed in *APOE3* mice. Among *APOE4* mice, there were also higher levels of Aβ immunoreactive load and FA-soluble Aβ42 in Female^CON^ relative to Male^CON^ mice but these were not significantly reduced in Female^MASC^ mice, suggesting that the protective effect of masculinization in *APOE4* mice may be limited to a subset of pathologies that includes mature amyloid plaques. Our finding of differential effects of masculinization on different Aβ fractions aligns with recent evidence that protective treatments can significantly reduce thioflavin-labeled but not Aβ immunoreactive pathologies in 5xFAD mice [61]. Although incomplete, these findings highlight a complex interplay between sex and *APOE* genotype in affecting the various pools of Aβ.

ApoE protein levels were also impacted by *APOE*, sex, and masculinization. Female^MASC^ *APOE4* mice presented intermediate phenotypes between Female^CON^ and Male^CON^ *APOE4* mice that moderated significant sex differences in levels of apoE protein in TBS- and detergent-soluble fractions of hippocampus. We previously found that the amount of apoE in each fraction is dependent on Aβ pathology, which in turn is age and brain region dependent [34, 53, 56, 62]. Essentially, with increasing amounts of Aβ, more apoE is found in the detergent- and FA-soluble extracts. These fractions likely represent apoE bound to Aβ, rather than changes in apoE processing. Indeed, in comparing Female^CON^ and Male^CON^ groups in this study, we found that detergent-soluble apoE levels were highest in female *APOE4* mice in the hippocampus and cortex. FA-soluble apoE was also highest in female *APOE4* mice in the cortex, but not in the hippocampus, which had likely reached saturation. These data are consistent with the higher Aβ levels observed in female *APOE4* mice, as we have shown before [53–55]. In terms of masculinization, Female^MASC^ *APOE3* mice had lower TBS-soluble levels of apoE and trends of higher detergent- and FA-soluble apoE levels in the hippocampus, and FA-soluble levels in the cortex in comparison to matched Female^CON^ mice. These results are consistent with higher TBS-soluble oAβ and higher Aβ immunohistochemical load in the subiculum associated with masculinization in *APOE3* mice. Therefore, one explanation is that apoE was binding to the higher amounts of deposited Aβ found in Female^MASC^ *APOE3* mice. In support of this possibility, our previous studies using treatments that target apoE levels and lipidation showed that when extracellular Aβ is lowered, apoE levels are increased in the TBS-soluble fraction and decreased in the FA-soluble fraction [62, 63]. In *APOE4* mice, apoE levels were lower in Female^MASC^ than Female^CON^ in the detergent- (hippocampus) and FA-soluble (cortex) extracts, which correspond to the trends in lower plaques. Thus, masculinization of *APOE4* females may have lowered Aβ pools, that in turn impacted apoE levels. Future studies should also evaluate the effects of masculinization on apoE levels and processing and corresponding relationships with Aβ levels.

*APOE* genotype significantly affected all outcomes. Consistent with prior work in AD mice [53–55], *APOE4* genotype was associated with increased levels of Aβ pathologies, especially in females. We also observed generally higher apoE levels in hippocampal homogenates from *APOE4* mice, especially females, effects that were more robust than in our previous study [53]. Unexpectedly, *APOE3* was often associated with results in Female^MASC^ mice that qualitatively differed from *APOE4*. In particular, the Female^MASC^ group in *APOE4* mice were generally intermediate between Female^CON^ and Male^CON^ groups, whereas Female^MASC^ *APOE3* mice often showed a distinct phenotype from both Female^CON^ and Male^CON^ mice. Specifically, Female^MASC^ *APOE3* mice showed levels of Aβ immunoreactive load and soluble Aβ and apoE in hippocampal fractions that were higher than both the Female^CON^ and Male^CON^ groups. The factor(s) underlying this *APOE*-dependent effect of perinatal masculinization on AD-related measures in uncertain, however it is noteworthy that *APOE* genotype also appeared to affect the extent of sexual dimorphisms and the responses to masculinization. We observed more robust sex differences in *APOE4* mice not only in AD-related endpoints, but also in anogenital distance and body weight. Further, perinatal masculinization yielded a stronger male-like phenotype for anogenital distance in *APOE3* versus *APOE4* Female^MASC^ mice, suggesting *APOE*-associated effects on systemic responsiveness to sex hormones. We were unable to assess SDN-MPOA and sexual behaviors in the experimental mice, data that would have further informed the relationship between *APOE* and sexual differentiation. Interestingly, findings in both humans [64, 65] and mice [66] report significant differences in fecundity in *APOE3* versus *APOE4* carriers. *APOE* genotype also affects the relationships between sex hormones and various neural and AD-related outcomes in humans [67, 68] and mice [46, 69, 70]. Thus, our findings build upon an accumulating literature that *APOE* genotype interacts with biological sex in diverse manners, perhaps including sex hormone responsiveness, that affect sex differences in AD and presumably therapeutic strategies.

The potential mechanisms driving these observations are unclear but reasonably may include microglia, which both contribute to the process of sexual differentiation in the brain [71] and exhibit transcriptomic and functional sex differences in adulthood [72, 73]. The established protective roles of microglia against mature plaques [58] show significant sexual dimorphisms in humans [74] and AD mouse models [75] that can contribute to elevated Aβ accumulation in females [76]. Interestingly, female microglia transcriptomic profiles [77, 78], reactive phenotypes [55], and impaired amyloid plaque interactions [59] can be further impacted by *APOE4* genotype. Our Iba-1 immunohistochemical data confirm prior findings of an *APOE4*-associated increase in microglial abundance [46]. We also observed two sex-dependent relationships. First, in *APOE4* EFAD mice only, microglial density was highest in the Female^CON^ group; this effect was not significantly moderated in the Female^MASC^ group. Second, specifically in *APOE3* EFAD mice, microglial responsive morphological phenotype was significantly increased in both female groups relative to the Male^CON^ group, with the highest levels in the Female^MASC^ group. Similar patterns in the *APOE3* groups were observed in Aβ load, oligomeric Aβ42, and detergent soluble apoE, which may indicate a relationship between sexual dimorphism, apoE-Aβ, interactions, and microglial responses in *APOE3* mice. Additional investigation is needed to further elucidate the possible contributions of microglia and other cells types to the effects of masculinization on development of AD pathologies.

There are some limitations to this study. First, as with most transgenic mouse models of AD, the EFAD model emphasizes Aβ over tau pathologies. Although the results demonstrate significant relationships among sex, *APOE* genotype, and sexual differentiation in regulation of Aβ, evidence from human studies indicates that the female sex x *APOE4* interaction is more important in the context of phosphorylated tau [10, 18]. Second, the *Thy1* promoter utilized in the EFAD and several other transgenic mouse models contains a putative estrogen response element [79]. Although this may present challenges in studies of sex differences, it does not explain observed differences nor undermine the use of these mouse models [80]. Third, while the employed perinatal masculinization approach is appropriate and well established, its value lies in a robust modulation of sexual differentiation to demonstrate concepts rather than modeling nuanced differences that are thought to contribute to the spectrum of relative masculinity – femininity within sexes that characterizes human populations.

In summary, this study presents important new findings that inform on the underlying mechanisms contributing to the interaction between female sex and *APOE4* genotype in driving AD risk. Contributions of the organizational effects of sex hormones to AD sex differences have been suggested previously [1, 81] and are supported by compelling but limited findings [32, 33, 82]. The current findings support a significant but limited role of sexual differentiation in vulnerability to AD pathogenesis, suggesting that a relatively more masculine phenotype may partially protect females from specific Aβ pathologies, particularly in the presence of *APOE4*. It is noteworthy that experimental masculinization typically yielded intermediate phenotypes rather than complete rescue of female biases on AD-relevant outcomes, which indicate only a partial effect. The findings complement prior work that collectively suggest sexual dimorphisms in AD likely involve an interplay between sex chromosomes [83, 84], organizational effects of sex hormones during development, and activational effects of sex hormones in adulthood as well as their loss during normal age-related hormone depletion [1]. Continued investigation of issue is essential for understanding the relationships among sex, *APOE*, and AD and promises to promote the development of precision-medicine interventions.

**Table 1:** Statistical analyses associated with Figs. 2, 3, 4, 5, 6 and 7

Figure	Main Effects	APOE3 post hoc Comparisons	APOE4 post hoc Comparisons	Between APOE post hoc Comparisons
2 A	Perinatal Tx Group: *F*_(2,83)_ = 289.6; *p* < 0.0001 *APOE* X Perinatal Tx Group: *F*_(2,83)_ = 11.7; *p* < 0.001	Male^CON^ vs. Female^CON^: *p* < 0.0001 Female^CON^ vs. Female^MASC^: *p* < 0.0001	Male^CON^ vs. Female^CON^: *p* < 0.0001 Male^CON^ vs. Female^MASC^: *p* < 0.0001 Female^CON^ vs. Female^MASC^: *p* < 0.0001	*APOE3* Male^CON^ vs. *APOE4* Male^CON^: *p* < 0.01 *APOE3* Female^MASC^ vs. *APOE4* Female^MASC^: *p* < 0.001
2B	*APOE*: *F*_(1,88)_ = 13.0; *p* < 0.001 Perinatal Tx Group: F_(2,88)_ = 40.8; *p* < 0.0001 *APOE* X Perinatal Tx Group: *F*_(2,83)_ = 12.7; *p* < 0.001	Male^CON^ vs. Female^CON^: *p* < 0.05	Male^CON^ vs. Female^CON^: *p* < 0.0001 Male^CON^ vs. Female^MASC^: *p* < 0.0001 Female^CON^ vs. Female^MASC^: *p* < 0.0001	*APOE3* Male^CON^ vs. *APOE4* Male^CON^: *p* < 0.0001 *APOE3* Female^MASC^ vs. *APOE4* Female^MASC^: *p* = 0.053
3B	*APOE*: *F*_(1,48)_ = 54.6; *p* < 0.0001 Perinatal Tx Group: F_(2,48)_ = 5.7; *p* < 0.01		Male^CON^ vs. Female^CON^: *p* < 0.05	*APOE3* Male^CON^ vs. *APOE4* Male^CON^: *p* < 0.01 *APOE3* Female^CON^ vs. *APOE4* Female^CON^: *p* < 0.0001 *APOE3* Female^MASC^ vs. *APOE4* Female^MASC^: *p* < 0.0001
3 C	*APOE*: *F*_(1,47)_ = 48.8; *p* < 0.0001 Perinatal Tx Group: *F*_(2,47)_ = 3.8; *p* < 0.05		Male^CON^ vs. Female^CON^: *p* < 0.05	*APOE3* Male^CON^ vs. *APOE4* Male^CON^: *p* < 0.01 *APOE3* Female^CON^ vs. *APOE4* Female^CON^: *p* < 0.0001 *APOE3* Female^MASC^ vs. *APOE4* Female^MASC^ *p* < 0.0001
4B	*APOE*: *F*_(1,48)_ = 18.4; *p* < 0.0001 Perinatal Tx Group: *F*_(2,48)_ = 12.5; *p* < 0.0001	Male^CON^ vs. Female^MASC^: *p* < 0.0001 Female^CON^ vs. Female^MASC^: *p* < 0.05	Male^CON^ vs. Female^MASC^: *p* < 0.05	*APOE3* Male^CON^ vs. *APOE4* Male^CON^: *p* < 0.01 *APOE3* Female^CON^ vs. *APOE4* Female^CON^: *p* < 0.01
4 C	*APOE*: F_(1,48)_ = 29.3; *p* < 0.0001 Perinatal Tx Group: F_(2,48)_ = 8.9; *p* < 0.001 *APOE* X Perinatal Tx Group: F_(2,48)_ = 2.4; *p* = 0.10	Male^CON^ vs. Female^MASC^: *p* < 0.01 Female^CON^ vs. Female^MASC^: *p* = 0.054	Male^CON^ vs. Female^CON^: *p* < 0.05 Male^CON^ vs. Female^MASC^: *p* = 0.07	*APOE3* Male^CON^ vs. *APOE4* Male^CON^: *p* < 0.01 *APOE3* Female^CON^ vs. *APOE4* Female^CON^: *p* < 0.0001
5 A	Perinatal Tx Group: *F*_(2,50)_ = 3.8; *p* < 0.05	Male^CON^ vs. Female^MASC^: *p* < 0.05		
5B	*APOE*: *F*_(1,53)_ = 57.4; *p* < 0.0001 Perinatal Tx Group: *F*_(2,53)_ = 9.8; *p* < 0.001 *APOE* X Perinatal Tx Group: *F*_(2,53)_ = 10.1; *p* < 0.001		Male^CON^ vs. Female^CON^: *p* < 0.0001 Male^CON^ vs. Female^MASC^: *p* < 0.0001	*APOE3* Female^CON^ vs. *APOE4* Female^CON^: *p* < 0.0001 *APOE3* Female^MASC^ vs. *APOE4* Female^MASC^: *p* < 0.0001
6 A	Perinatal Tx Group: *F*_(2,52)_ = 4.4; *p* < 0.05 *APOE* X Perinatal Tx Group: *F*_(2,52)_ = 7.7; *p* < 0.01	Male^CON^ vs. Female^MASC^: *p* < 0.01 Female^CON^ vs. Female^MASC^: *p* < 0.05	Male^CON^ vs. Female^CON^: *p* < 0.01	*APOE3* Male^CON^ vs. *APOE4* Male^CON^: *p* < 0.05 *APOE3* Female^MASC^ vs. *APOE4* Female^MASC^: *p* < 0.05
6B	*APOE*: *F*_(1,53)_ = 5.5; *p* < 0.001 Perinatal Tx Group: *F*_(2,53)_ = 5.3; *p* < 0.01 *APOE* X Perinatal Tx Group: *F*_(2,53)_ = 5.3; *p* < 0.01	Male^CON^ vs. Female^MASC^: *p* < 0.01	Male^CON^ vs. Female^CON^: *p* < 0.05 Female^CON^ vs. Female^MASC^: *p* < 0.05	*APOE3* Male^CON^ vs. *APOE4* Male^CON^: *p* < 0.01 *APOE3* Female^CON^ vs. *APOE4* Female^CON^: *p* < 0.001
6 C	*APOE*: *F*_(1,53)_ = 3.2; *p* = 0.08 Perinatal Tx Group: *F*_(2,53)_ = 6.1; *p* < 0.01	Male^CON^ vs. Female^MASC^: *p* < 0.05 Female^CON^ vs. Female^MASC^: *p* = 0.07		
7B	*APOE*: *F*_(1,51)_ = 40.9; *p* < 0.0001			*APOE3* Male^CON^ vs. *APOE4* Male^CON^: *p* < 0.0001 *APOE3* Female^CON^ vs. *APOE4* Female^CON^: *p* < 0.0001 *APOE3* Female^MASC^ vs. *APOE4* Female^MASC^ *p* < 0.05
7 C	*APOE*: *F*_(1,51)_ = 42.3; *p* < 0.0001			*APOE3* Male^CON^ vs. *APOE4* Male^CON^: *p* < 0.001 *APOE3* Female^CON^ vs. *APOE4* Female^CON^: *p* < 0.0001 *APOE3* Female^MASC^ vs. *APOE4* Female^MASC^ *p* < 0.01
7D	*APOE*: *F*_(1,51)_ = 101.5; *p* < 0.0001 Perinatal Tx Group: *F*_(2,51)_ = 10.3; *p* < 0.001 *APOE* X Perinatal Tx Group: *F*_(2,51)_ = 2.9; *p* = 0.06		Male^CON^ vs. Female^CON^: *p* < 0.0001 Male^CON^ vs. Female^MASC^: *p* < 0.01	*APOE3* Male^CON^ vs. *APOE4* Male^CON^: *p* < 0.001 *APOE3* Female^CON^ vs. *APOE4* Female^CON^: *p* < 0.0001 *APOE3* Female^MASC^ vs. *APOE4* Female^MASC^ *p* < 0.0001
7E	*APOE*: *F*_(1,51)_ = 139.2; *p* < 0.0001 Perinatal Tx Group: *F*_(2,51)_ = 9.5; *p* < 0.001	Male^CON^ vs. Female^CON^: *p* < 0.05 Male^CON^ vs. Female^MASC^: *p* < 0.001		*APOE3* Male^CON^ vs. *APOE4* Male^CON^: *p* < 0.0001 *APOE3* Female^CON^ vs. *APOE4* Female^CON^: *p* < 0.0001 *APOE3* Female^MASC^ vs. *APOE4* Female^MASC^ *p* < 0.0001

## Supplementary Information

Supplementary Material 1.

## Data Availability

All data supporting the findings of this study are available within the paper and its Supplementary Materials. The authors will provide raw data files upon reasonable request.
