# Supplementary material for: Effects of sexual differentiation and APOE genotype on the development of Alzheimer-related pathology in female EFAD mice
Source: Biol Sex Differ. 2026 Aug 12;17:178. doi: 10.1186/s13293-026-00967-w (PMC13746649; doi:10.1186/s13293-026-00967-w)
Supplement: Supplementary file 1 [file 13293_2026_967_MOESM1_ESM.docx]

**Supplementary materials for *Effects of sexual differentiation and APOE genotype on the development of Alzheimer-related pathology in female EFAD mice* by Christensen et al.**

**Table S1.** Statistical analyses associated with Supplementary Figure 2.

| **Figure** | **Test** | **Effects** | ***post hoc* Comparisons** |
| --- | --- | --- | --- |
| S2A | One-way ANOVA | *F*_(2,9)_= 16.7; *p*<0.001 | Male^CON^ vs Female^CON^: *p*<0.01  Female^CON^ vs Female^MASC^; *p*<0.01 |
| S2B | Two-tailed *t*-test | *t* _(10)_ = 24.9; *p*<0.0001 |  |

**Table S2.** Statistical analyses associated with Supplementary Figures 3, 4.

| **Figure** | **Main Effects** | ***APOE3 post hoc* Comparisons** | ***APOE4 post hoc* Comparisons** | **Between *APOE* *post hoc* Comparisons** |
| --- | --- | --- | --- | --- |
| S3A | *APOE*: *F*_(1,52)_= 3.2; *p*=0.08  Perinatal Tx Group: *F*_(2,52)_= 7.3; *p*<0.01  *APOE* X Perinatal Tx Group: *F*_(2,52)_= 3.8; *p*<0.05 | Male^CON^ vs Female^MASC^: *p*<0.05  Female^CON^ vs Female^MASC^: *p*<0.05 | Male^CON^ vs Female^CON^: *p*<0.01  Male^CON^ vs Female^MASC^: *p*<0.05 | *APOE3* Female^CON^ vs *APOE4* Female^CON^: *p*<0.01 |
| S3B | *APOE*: *F*_(1,53)_= 21.0; *p*<0.0001  Perinatal Tx Group: *F*_(2,53)_= 13.6; *p*<0.0001  *APOE* X Perinatal Tx Group: *F*_(2,53)_= 3.6; *p*<0.05 | Male^CON^ vs Female^MASC^: *p*=0.10 | Male^CON^ vs Female^CON^: *p*<0.0001  Male^CON^ vs Female^MASC^: *p*<0.0001 | *APOE3* Female^CON^ vs *APOE4* Female^CON^: *p*<0.0001  *APOE3* Female^MASC^ vs *APOE4* Female^MASC^: *p*<0.01 |
| S4A | *APOE*: *F*_(1,52)_= 18.1; *p*<0.0001  Perinatal Tx Group: *F*_(2,52)_= 8.9; *p*<0.001  *APOE* X Perinatal Tx Group: *F*_(2,52)_= 5.9; *p*<0.01 |  | Male^CON^ vs Female^CON^: *p*<0.0001  Male^CON^ vs Female^MASC^: *p*=0.10  Female^CON^ vs Female^MASC^: *p*<0.05 | *APOE3* Female^CON^ vs *APOE4* Female^CON^: *p*<0.0001 |
| S4B | *APOE*: *F*_(1,53)_= 23.6; *p*<0.0001  Perinatal Tx Group: *F*_(2,53)_= 3.5; *p*<0.05  *APOE* X Perinatal Tx Group: *F*_(2,53)_= 6.4; *p*<0.01 |  | Male^CON^ vs Female^CON^: *p*<0.01  Male^CON^ vs Female^MASC^: *p*<0.001 | *APOE3* Female^CON^ Female vs *APOE4* Female^CON^: *p*<0.001  *APOE3* Female^MASC^ vs *APOE4* Female^MASC^: *p*<0.0001 |
| S4C | *APOE*: *F*_(1,53_)= 14.3; *p*<0.001  Perinatal Tx Group: *F*_(2,53)_= 8.5; *p*<0.001  *APOE* X Perinatal Tx Group: *F*_(2,53)_= 10.9; *p*<0.001 |  | Male^CON^ vs Female^CON^: *p*<0.0001  Female^CON^ vs Female^MASC^: *p*<0.0001 | *APOE3* Female^CON^ vs *APOE4* Female^CON^: *p*<0.0001 |

**Supplemental Figure 1**

**Supplemental Figure 1. Schematic of brain processing.** (Left panel) Analyses of the sexually dimorphic nucleus of medial preoptic area (SDN-MPOA) utilized brain sections in the coronal plane and assessed the nucleus area as determined by clusters of cells labeled with the neuron-specific marker NeuN (black oval, lower left panel) located in the MPOA brain region (boxed area, middle left panel) from bregma coordinates 0.2 – 0.1 mm. (Right panel) Histochemical analyses of the hippocampal formation used brain sections in the horizontal plane and examined both the hippocampus CA1 and subiculum subregions (boxed area, middle right panel) across the interaural coordinate range 1.2 – 2.8 mm for markers of plaque pathology and microglia. This figure was created in BioRender. Pike, C. (2026) https://BioRender.com/oy4otrb.

**Supplemental Figure 2**

**Supplemental Figure 2. Effects of experimental treatments on neural and behavioral measures of sexual dimorphism.** Measures of sexual differentiation were assessed at age 3 months in *APOE3* and *APOE4* EFAD non-carrier littermates of the experimental animals. (A) The size of the sexually dimorphic area of the medial preoptic area as determined by area measurement. (B) Sexual behavior in female groups primed with estradiol and in the presence of a male animal as determined by lordosis quotient. Analyses were made from two or more of the following treatment groups: control males (Male^CON^, open bars), masculinized females (Female^MASC^, hatched bars), and control females (Female^CON^, solid green bars). Data show mean ± SEM; individual data points are indicated by solid circles. Details of statistical analyses are shown in Table S1. Brackets denote significant between group differences: ** *p*< 0.01, **** *p*< 0.0001.

**Supplemental Figure 3**

**Supplemental Figure 3. Effects of experimental treatments on levels of oligomeric and insoluble β-amyloid in the cerebral cortex.** Levels of the highly amyloidogenic Aβ42 species of β-amyloid protein were measured by ELISA in cerebrocortical homogenates. (A) Oligomeric Aβ42 (oAβ) was measured in the soluble fraction, and (B) relatively insoluble Aβ42 was measured in the formic acid soluble fraction from control males (Male^CON^, open bars), masculinized females (Female^MASC^, hatched bars), and control females (Female^CON^, solid green bars). Data show mean ± SEM; data points from individual mice are indicated by solid circles. Details of statistical analyses are shown in Table 1. Brackets denote significant between group differences: * *p*< 0.05; ** *p*< 0.01; **** *p*< 0.0001.

**Supplemental Figure 4**

**Supplemental Figure 4. Effects of experimental treatments on levels of apoE protein in the cerebral cortex.** Levels of apoE were measured by ELISA in cerebrocortical homogenates extracted into (A) TBS-soluble fractions, (B) detergent (Triton-X100)-soluble fractions, and (C) relatively insoluble formic acid-soluble fractions from control males (Male^CON^, open bars), masculinized females (Female^MASC^, hatched bars), and control females (Female^CON^, solid green bars). Data show mean ± SEM; data points from individual mice are indicated by solid circles. Details of statistical analyses are shown in Table 1. Brackets denote significant between group differences: * *p*< 0.05; ** *p*< 0.01; *** *p*< 0.001; **** *p*< 0.0001.
